# Development of a Danish Language Version of the Manchester Foot Pain and Disability Index: Reproducibility and Construct Validity Testing

**DOI:** 10.1155/2013/284903

**Published:** 2013-03-06

**Authors:** Christian K. Pedersen, Bente Danneskiold-Samsøe, Adam P. Garrow, Eva E. Wæhrens, Henning Bliddal, Robin Christensen, Else Marie Bartels

**Affiliations:** ^1^The Parker Institute, Department of Rheumatology, Copenhagen University Hospital Bispebjerg and Frederiksberg, Nordre Fasanvej 57, 2000 Frederiksberg, Denmark; ^2^Medicines Evaluation Unit, The Langley Building, Southmoor Road, Wythenshawe, Manchester M23 9QZ, UK

## Abstract

*Introduction*. The Manchester Foot Pain and Disability Index (MFPDI) is a 19-item questionnaire for the assessment of disability caused by foot pain. The aim was to develop a Danish language version of the MFPDI (MFPDI-DK) and evaluate its reproducibility and construct validity. *Methods*. A Danish version was created, following a forward-backward translation procedure. A sample of 84 adult patients with foot pain was recruited. Participants completed two copies of the MFPDI-DK within a 24- to 48-hour interval, along with the Medical Outcomes Study Short Form 36 (SF-36), and a pain Visual Analog Scale (VAS). Reproducibility was assessed using the intraclass correlation coefficient (ICC) and 95% limits of agreement (Bland-Altman plot). Construct validity was evaluated with Pearson's Rho, using a priori hypothesized correlations with SF-36 subscales and VAS_mean_. *Results*. The MFPDI-DK showed very good reliability with an ICC of 0.92 (0.88–0.95). The 95% limits of agreement ranged from −6.03 to 6.03 points. Construct validity was supported by moderate to very strong correlations with the SF-36 physical subscales and VAS_mean_. *Conclusion*. The MFPDI-DK appears to be a valid and reproducible instrument in evaluating foot-pain-related disability in Danish adult patients in cross-sectional samples. Further research is needed to test the responsiveness of the MFPDI-DK.

## 1. Introduction 

Foot pain is among the most frequent musculoskeletal complaints in the western world. Studies show that approximately 30% of people older than 65 years have experienced recent foot pain [1–6]. Mølgaard et al. verified this prevalence in a Danish population-based study, where 30.4% reported foot pain within the previous month [7]. Foot pain can be disabling in many ways. It may lead to impaired locomotion, which in turn leads to a reduced ability to perform activities of daily living and an increased risk of injury causing falls. Furthermore, foot pain in the elderly leads to an increased risk of depression and a reduced health-related quality of life (HRQoL) [1, 3, 8–12]. Region or disease-specific Patient-Reported Outcomes (PROs) are becoming an important method to report the severity of a functional problem, measure the effect of treatment modalities, and obtain comparable clinical-scientific results. There is a general consensus that PROs should serve as the gold standard when assessing musculoskeletal conditions, where the patient's perspective and effects on HRQoL is the primary interest [13–17].

Internationally, there are a number of foot-specific PROs available. However, a Danish version of a validated, foot-specific PRO is yet to be developed. The need for valid and reliable PROs was emphasized in a study by Marshall et al. [18], which demonstrated that the use of unpublished measurement instruments was more likely to report positive effects of treatment than clinical trials using published instruments. 

Following consideration of the most commonly applied PROs, we chose the British-developed Manchester Foot Pain and Disability Index (MFPDI) [19]. MFPDI fitted our requirements well: the MFPDI is readily implementable due to small cross-cultural differences between Denmark and Britain, and it is validated in patients of different ages, suffering from a variety of foot disorders. MFPDI is, furthermore, a generic tool which can be applied in many different clinical and scientific settings in Denmark [19–21]. Additionally, translations into other European languages have been made successfully [6, 22–24], making the tool applicable for trans-national comparisons of results.

 The MFPDI consists of a total of 19 items [6, 19, 22]. These are related to pain intensity (five items), functional limitation (10 items), personal appearance (two items), and performance of work and leisure activities (two items). These two last items are in the original version excluded if the patient is of retirement age. All items begin with the phrase “Because *of pain in my feet…*” followed by a statement related to foot problems. All items are rated on a three-category ordinal scale, using one of the following responses: “None of the time” (score = 0), “On some days” (score = 1), and “On most/every day” (score = 2) [19]. A total score is achieved by summing the ordinal item scores, thus making the scoring interval 0–38 points (0–34 if items 18 and 19 are excluded). The questionnaire is therefore short enough not to be a burden to answer but covers all important aspects of experiencing foot pain.

The aim of the present study was to develop a Danish version of the MFPDI (MFPDI-DK) and subsequently examine its reproducibility as well as construct validity. 

## 2. Methods

The properties of the original questionnaire had already been proven valid in a British population, which as a Northern European culture may be considered culturally similar to Denmark [19]. It was therefore assumed that the content of the questionnaire in its original form would also be cross-culturally valid in Danish. Consequently, it was decided to carry out a construct validation combined with an examination of reproducibility. Permission to translate and use the questionnaire in this study was granted by ISIS Innovation, Oxford, UK (http://www.isis-innovation.com/), and ISIS guide lines were followed and were in accordance with the ones outlined below. 

### 2.1. Translation and Pilot Version Testing

**Figure 1:**
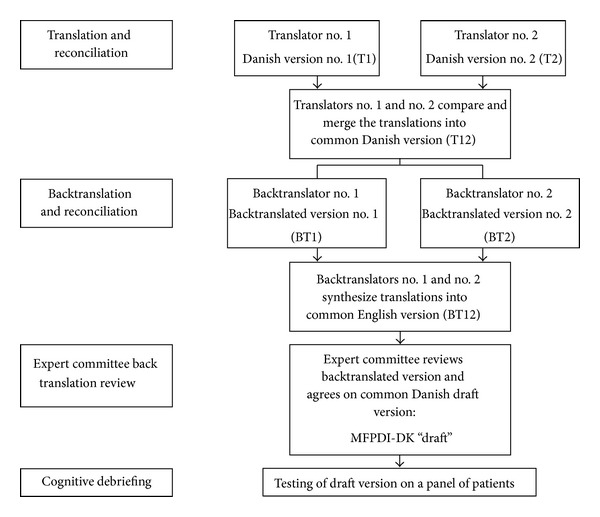
Translation process. Translation followed the guidelines of translation specified in “Principles of good practice for the translation and cross-cultural adaption process for patient-reported outcomes (PRO) measures” by the ISPOR Task Force.

Translation followed the guidelines of translation specified in “Principles and good practice for the translation and cross-cultural adaptation process for PRO measures” by the ISPOR Task Force [25]. The translation process is shown in Figure 1. Two translators independently produced a translated version of the English version into Danish (T1 and T2). T1 and T2 were compared and reconciled, producing the first Danish version (T12). Then two bilingual translators, naïve to the original questionnaire, produced two English translations of T12 (BT1 and BT2), which were also compared and reconciled into a common version (BT12). An expert committee consisting of all translators (T1, T2, BT1, and BT2), one content expert, and two senior researchers from the Parker Institute, all fluent in Danish and English, reviewed the backtranslation, comparing it to the original MFPDI. Items showing discrepancies were discussed and revised, producing a Danish draft version (MFPDI-DK “draft”).

The draft version was tested on a group of five patients recruited from the out-patient department of Rheumatology at Frederiksberg Hospital. Subsequently, all items were discussed in a semistructured interview. Following the interviews, the expert committee discussed the results and carried out the last revisions and proof reading. 

### 2.2. Sample Size and Participants

We wished to obtain a power of 90% which decided the number of patients necessary in the study. In a two one-sided test (TOST) analyses for additive equivalence of paired means, with bounds −1 and 1 for the mean difference in the MFPDI-DK and a significance level of 0.05, assuming a mean difference of 0, a common standard deviation of 3.7, and correlation of 0.75, a sample size of 76 pairs was required to obtain a power of at least 90% (the actual power was 90.2%). Based on this, it was decided to include at least 80 patients in total in order to have a reasonable power with a narrow 95% confidence interval (−1 to +1) around the test-retest estimate of MFPDI-DK. Participants were volunteers recruited from various sources: local members of the *National Association of State-Registered Chiropodists (LaSF)*, the outpatient clinic of rheumatology at *Frederiksberg Hospital*, and the Orthopedic Surgical Ward at the private hospital *Aleris Hamlet*. Patients showing up for scheduled appointments with their clinician or chiropodist were informed about the study and asked to participate. Inclusion criteria were reports of foot pain within the last month. Exclusion criteria were inability to read Danish and inability to walk household distances. Patients with chronic widespread pain were also excluded. Written and verbal information was provided to all participants.

### 2.3. Study Design

The study was designed as a test-retest study. Upon accepting to participate, patients received an envelope containing a sheet of information about the project, a general characteristics questionnaire, the Danish version of the Medical Outcomes Study Short Form 36 (SF-36) [26], two MFPDI-DK questionnaires, and a stamped addressed envelope for return of the completed questionnaires. The two MFPDI-DK questionnaires were to be used in the test-retest analysis, and patients were instructed to complete one on the same day and the other 24–48 hours later.

### 2.4. Statistical Analysis

#### 2.4.1. Construct Validity

The COSMIN checklist (COnsensus-based Standards for the selection of health status Measurement INstruments) [27] defines construct validity as “*the degree to which the scores of a (HR)-PRO instrument are consistent with hypotheses (for instance with regard to …relationships to scores of other instruments…) based on the assumption that the (HR)-PRO instrument validly measures the construct to be measured*”. In this case, the other instruments were SF-36 [26] and a universal VAS scale addressing the mean level of pain within the previous month [28]. Construct validity was assessed by the calculation of Pearson's correlation coefficient (Rho). It was hypothesized a priori that a strong association would be found between MFPDI-DK and SF-36 Physical Functioning (PF) and Physical Component Score (PCS) subscales, a moderate to strong correlation between MFPDI-DK and SF-36 Bodily Pain (BP), Role-Physical (RF), Vitality (VT), General Health (GH), and VAS_mean_, and weak to moderate correlations for the MFPDI-DK and mental subscales of the SF-36 which were expected.

#### 2.4.2. Reproducibility

In order to investigate the reproducibility of the Danish MFPDI, we examined the test-retest reliability, as well as agreement between repeated scores, by asking participants to complete two copies of the MFPDI-DK within a time interval of 24–48 hours. Following the Guidelines for Reporting Reliability and Agreement Studies (GRRAS) [29], agreement is the degree to which repeated scores obtained from the same patient are identical (given that the patient is in a stable condition), while reliability is the ability to differentiate between subjects or objects. Reliability was evaluated using the intraclass correlation coefficient (two-way mixed-effect model, absolute agreement, single measure, and ICC), where an ICC value of 0 is synonymous with no correlation of repeated scores and 1 with perfect correlation. For this study, an ICC of at least 0.75 was considered acceptable. To evaluate the agreement between scores, the difference between the repeated MFPDI-DK scores against their mean was illustrated using a Bland-Altman plot with 95% limits of agreement [30].

All statistical analyzes were performed using *IBM SPSS statistics 19* for Windows. 

## 3. Results

### 3.1. Translation

**Figure 2:**
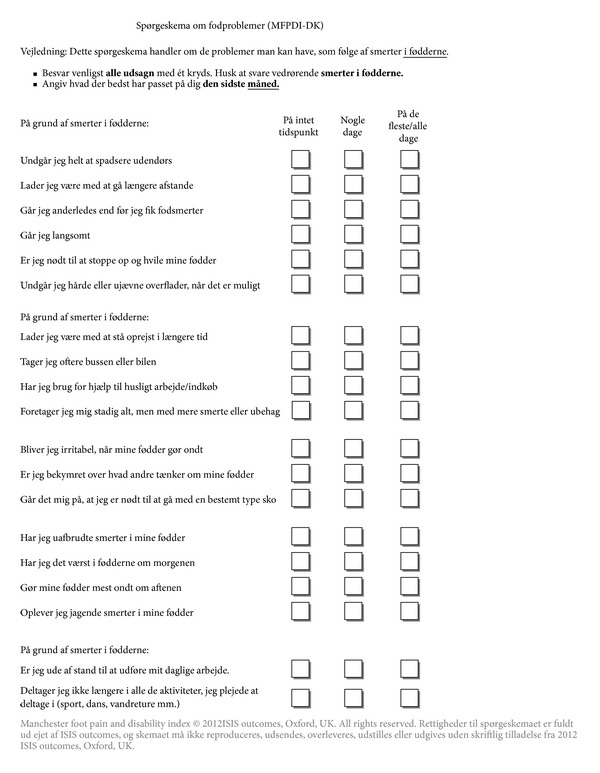
MFPDI-DK. Compared to the original, the Danish version of the Manchester Foot Pain and Disability Index has been altered slightly, following cognitive debriefing and discussion in the expert committee. Items no. 18 and no. 19 are included regardless of the patient's age, and the “not applicable” boxes have been removed. The heading means “Questionnaire about foot problems (MFPDI-DK)” and was chosen to give the patients a clear idea of what the questionnaire is about. The instruction section reminds patients of ticking one box for each statement, and that the questionnaire is concerned with *foot pain* within the previous *month*. Similar to the original, the subheadings function to remind the patients of answering regarding their feet. Notice that ISIS outcome has the copyright on this questionnaire and have to give written permission prior to use.

The Danish version of the MFPDI is shown in Figure 2. Translation was relatively straight-forward without major discrepancies. However, items no. 1 (*…I avoid walking outside at all*) and no. 3 (*I don't walk in a normal way*) showed some translational difficulties. Item 1: “*because of pain in my feet I avoid walking outside at all*” was a challenge, since the commonly applied term for “walking” (“at gå”) also means “to go.” This could lead to readers thinking that the item referred to the act of going outside, that is, leaving their home. After some discussion, the word “spadsere” was chosen. This term is not commonly applied when referring to walking as a means of transportation (the meaning of the word is similar to “stroll”). However, it was the only way of manifesting the intended meaning of the item. Item 3: “…*I don't walk in a normal way*” was potentially confusing, since “normal” can refer both to patients' current walking style compared to peers or their walking style before the foot disorder debuted. It was therefore decided to change the wording into “*I walk in a different way than before I had foot pains*” to emphasize the intended meaning.

### 3.2. Cognitive Debriefing

Overall, the participants in the pilot group were very pleased with the questionnaire. They appreciated the one-page layout and how easily comprehensible the statements were, and they found the statements to be highly relevant to the aspects of foot disease, while at the same time being to the point and quick to complete. The cognitive debriefing followed by review in the expert panel brought about some further revisions. These are addressed below. 

Item 18: “… *I am unable to carry out my previous work,*” which in the English version is removed when the patient is of retirement age, was a matter of great discussion in the group. It was agreed that this item is relevant to all Danish patients, regardless of age and employment status, since it also covers the concept of “daily duties” or “chores”. This makes the item relevant to the senior population in Denmark who are generally very active. 

Item 19: “*…I no longer do all my previous activities (sport, dancing, hill-climbing, etc.)*” was also considered universally relevant, since most Danish pensioners have a hobby they attend. Based on the cognitive debriefing, it was therefore decided to include all 19 items, regardless of the patient's age. Furthermore, it was decided to remove the “not applicable” boxes and move the bottom sentence (*tick here when you have read all the statements on this page*) to the instructions at the top of the page. 

### 3.3. Participants

**Figure 3:**
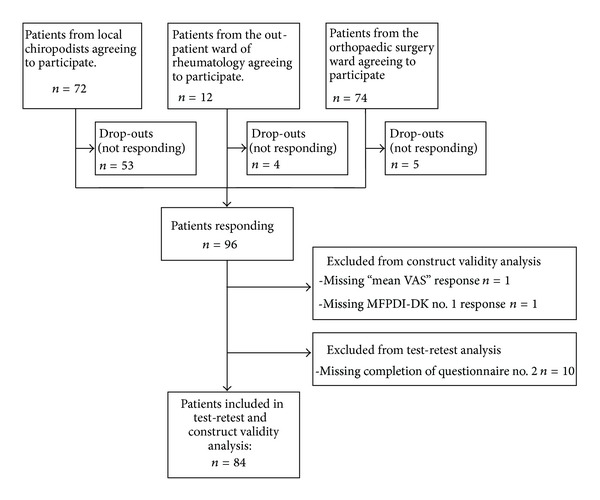
Participant Flow Chart showing the different sources of participants. 158 agreed to participate upon request from the clinician. 96 (60.8%) replied. 12 of these were excluded, leaving 84 participants in the final analysis.

A total of 158 questionnaire kits were handed out to patients with foot complaints agreeing to participate in the study. Responder rate was 60.8% (96 replies). 12 of the 96 repliers were excluded due to missing data in the form of not answering one or more questionnaires or not answering on VAS of pain (Figure 3). This left 84 participants (53.2% of original group). These 84 were our study group, and demographic characteristics of the group are shown in Table 1. The mean age was 57 years (range 14–84), and almost two thirds were women (61.9%).

### 3.4. Missing Data

*MFPDI*. At baseline, two items from a total of 84 patients × 19 items (0.1%) were missing. At re-test eight items from 84 patients × 19 items (0.5%) were missing, with three of these deriving from the same patient. A total score could be calculated for all subjects by allocating the participant's mean score to the missing items. Items no. 5, no. 12, and no. 19 had one missing reply on the second assessment, while item no. 16 had one missing reply on the first assessment. Item no. 10 was the only item with a missing reply on both assessments—these were both from the same patient. Items no. 11 and no. 13 had two missing replies on the second assessment, and none on the first.

*SF-36*. 19 items out of 84 patients × 36 items (0.5%) were missing. A total score could be calculated for all subjects on all subscales.

*VA* *S*_*mean*_. No missing items were reported for the VAS_mean_ scores in the study, since this was an exclusion criterion.

### 3.5. Scores and Correlation between Assessment Tools

Scores for the SF-36 subscales, VAS_mean_, and MFPDI-DK total and subscales are shown in Table 2.  Table 3 gives the Pearson correlation coefficients between MFPDI and SF-36 and VAS_mean_ scores. The correlation coefficients for the SF-36 subscales were expressed as negative values due to the inverse relationship between the instruments (SF-36: high score means better health, MFPDI: high score means worse condition). Total MFPDI-DK score was strongly associated with the SF-36* physical component score *(*r* = −0.66) and *physical functioning *(*r* = −0.62) subscales. The *role-physical *(*r* = −0.57), *bodily pain *(*r* = −0.52), *vitality *(*r* = −0.57), *general health *(*r* = −0.46), *mental component score *(*r* = −0.43), *social role functioning *(*r* = −0.48), and *mental health *(*r* = −0.49) subscales showed moderate correlations, while the *role-emotional* subscale showed weak correlation (*r* = −0.37). MFPDI-DK and VAS_mean_ scores showed moderate association (*r* = 0.42).

### 3.6. Reproducibility Testing

**Figure 4:**
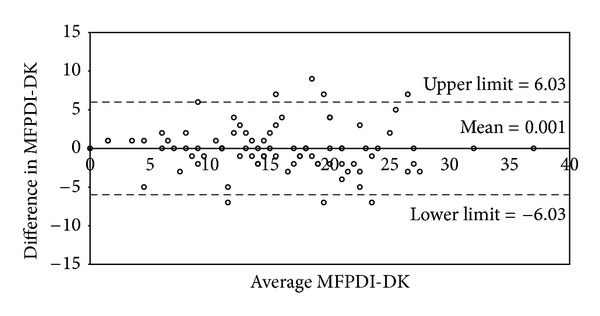
Bland-Altman plot illustrating agreement between repeated measurements of the MFPDI-DK. The difference between measurements is shown against the average MFPDI-DK score of the 84 participants. Mean difference = 0.001 (SD = 3.01), 95% limits of agreement = −6.03 : 6.03.

Test-retest assessment was carried out on 84 patients. The intraclass correlation coefficient (ICC) for the MFPDI total score was 0.92 (95% confidence interval, 0.88–0.95) (Table 4). The mean of the difference for repeated MFPDI scores was close to zero (Difference between the two measurements of MFPDI < 0.0012, SD = 3.01).  Figure 4 illustrates a Bland-Altman plot with the difference between paired sets of scores shown against their average MFPDI-DK score and the 95% limits of agreement for repeated MFPDI-DK assessment. The 95% limits of agreement ranged from −6.03 to 6.03.

## 4. Discussion

Until now, no Danish-language instrument for the assessment of foot-pain-related impact on physical functioning and health-related quality of life existed. The need for such an instrument is increasing, since region-specific disability scales have become important supplements to traditional assessment methods in clinical practice and clinical trials. Using translated versions of validated and acknowledged questionnaires will facilitate a standardization of outcome measurements, hereby strengthen the power of clinical research using PROs, and enable comparison of results worldwide. Our overall objective was to add a useful tool to facilitate the mapping of foot-pain-related impact on the daily life of Danish patients. Denmark is an ideal place for population-based studies due to the social security number system, and there is a proud tradition of conducting these on a large scale. A foot-specific, yet generic, questionnaire could be included in large-scale population studies, hereby adding to the current knowledge of foot-pain-related disability in various subgroups of the Danish population. Using validated instruments should always be pursued, and by applying internationally recognized instruments, it becomes possible to compare research results across borders. 

### 4.1. Translation

Compared to other languages, Danish have no significant variations in the use of words, and no regional differences exist in written language. Colloquialisms and ambiguous wordings were avoided to ensure that both younger and older people would have no difficulties in comprehending the statements. Translation of the questionnaire was overall straight forward with only a few minor difficulties, which were solved by discussions in the expert panel and by making contact to the developer of the MFPDI, Dr. Adam P. Garrow, who was involved early in the study to ensure that problematic items were translated in a manner that would reflect the intended meaning. Since the reverse translation was very much spot on compared to the original version and since the two reverse translations were more or less similar, we also concluded that there was a good conceptual equivalence. 

### 4.2. Study Population and Nonresponders

The participants were considered a representative sample of the Danish adult population. It was also assumed that they were culturally and educationally comparable, that is, that all participants were able to read and understand the questionnaire. The small amount of missing items combined with the high level of reproducibility between the repeated measurements of MFPDI scores suggests that this assumption is fair, and that the Danish translation was well comprehended by the participants. Figure 3 shows, that the nonresponder proportion of the original group was 39.2%. This is slightly higher than in the original study [19]. It becomes apparent that the main bulk of nonresponders are located in the chiropodist sample. This is likely explainable by the different modality of recruiting applied in this group. While at least one of the authors was personally responsible for recruiting in both the rheumatologic and orthopedic group, recruiting in the chiropodist group relied on the chiropodists themselves. Patients in this group were instructed to bring the full set of questionnaires home with them, complete them all on two consecutive days, and post everything in the mail. Therefore, the success rate of this group relied on the ability and effort of the chiropodist to instruct and guide the participants to actually complete and return the questionnaires. It is likely that a lot of the nonresponders are patients who accepted to participate to show good intentions but quickly lost their motivation when facing the task. It would have been preferable to have these patients complete most of the questionnaires in the clinic and only bring the MFPDI-DK for day two home with them, as it was the case in the orthopedic group. Sadly, this was not possible in the busy chiropodist clinics. 

### 4.3. Correlation between Assessment Tools

Construct validity of the Danish translation of the MFPDI was assessed by calculating correlations between the MFPDI-DK and the SF-36 subscales and VAS_mean_ scores. It was hypothesized that patients scoring poorly on one scale would also score poorly on the other. As expected, correlation was strongest for the physical subscales and weaker for the mental components. The correlations found are comparable to those found in a similar study by Kaoulla et al. [24]. MFPDI-DK and VAS_mean_ scores were moderately correlated (*r* = 0.42). This moderate correlation most likely reflects that the MFPDI-DK is a measure of disability caused by foot pain, that is, not a specific measure of pain alone. Patients suffering from severe pain are not necessarily highly limited in their daily function, and highly disabled patients are not necessarily affected by strong pain. 

### 4.4. Reproducibility

Test-retest analysis showed a high degree of reproducibility. Reliability was excellent with an ICC-score of 0.92 (Table 4). The Bland-Altman plot (Figure 4) demonstrated that the 95% level of agreement was spread across an interval of ±6 points. This relatively broad interval may reflect the generic nature of the questionnaire. A disease-specific instrument with questions aimed directly at concrete contexts of the disease makes it more likely that patients produce similar answers for repeated measurements, thereby leading to a narrower interval. The interval we obtained suggests that a difference of 7 points or more between two MFPDI-DK assessments is 95% certain to be a clinically significant one. However, it is a useful tool for a quick assessment of disability due to foot pain. Future research should investigate the responsiveness further. 

### 4.5. Limitations

Participation in the study was to some degree a burden for part of the patients due to the relatively large amount of effort required when filling in the questionnaires. Thus, in order to minimize the number of non-responders, the demographic characteristics questionnaire was made quite short in an attempt to minimize the amount of questions participants had to answer. The result of this gave few demographic details on the study group. The study design carries the risk of selection bias, since resourceful and healthier patients are more likely to register and complete the study than less resourceful and older patients. Regarding the large proportion of nonresponders in the chiropodist group, it would have been a major advantage to have the opportunity to send reminders to nonresponders. However, we were not able to collect contact information of the patients. Our study could be limited by the short time interval between measurements, which might be short enough for participants to recall their answers. The short interval was, on the other hand, considered a necessity to minimize drop-outs and to make certain that the patients' condition had not changed. Due to the relatively large amount of questions to complete, it is though the authors' opinion that the risk of exact recollection of items was small. 

Each question in the questionnaire has equal rating, and one may consider if each question has the same importance. This problem is to some degree eradicated by the number of questions in each subscale, and we therefore recommend that the total score is used to give the best assessment of the patient's condition due to foot pain.

## 5. Conclusion

The results of this study indicate that the Danish version of the MFPDI is a valid instrument for assessing disability caused by foot pain in Danish adults. The ability of the MFPDI-DK in measuring clinical effects of interventions should be explored in future research. 

## Figures and Tables

**Table 1:** Basic demographic characteristics of the study group (*n* = 84).

Age (years)	
Mean (SD)	57 (15.5)
Range: min–max	14–84
Age groups (years, *n* (%))	
≤50	27 (32.1%)
51–60	19 (22.6%)
61–70	20 (23.8%)
71–80	16 (19.1%)
>80	2 (2.4%)
Sex (*n*(%))	
Female	52 (61.9%)
Male	32 (38.1%)
Education: total years after public school (%)	
<2 years	8 (9.5%)
2–4 years	33 (39.3%)
5-6 years	17 (51.5%)
>6 years	33 (39.3%)
Missing response	1 (1.2%)

**Table 2:** MFPDI, VAS, and SF-36 scores.

	Mean	SD	Median	Range
MFPDI-DK day 1 (*n* = 84)				
Total score	15.8	7.4	15.0	0–37
Physical subscale	8.8	4.9	9.0	0–19
Pers. appearance subscale	0.9	1.1	1.0	0–4
Pain subscale	4.5	2.3	5.0	0–10
Work/leisure	1.6	1.1	2.0	0–4
MFPDI-DK day 2 (*n* = 84)				
Total score	15.8	7.3	15.5	0–37
Physical subscale	8.6	4.9	9.0	0–19
Pers. appearance subscale	0.9	1.1	0.0	0–4
Pain subscale	4.7	2.2	5.0	0–10
Work/leisure	1.7	1.2	2.0	0–4
MFPDI-DK_mean_ (*n* = 84)				
Total score	15.8	7.4	15.0	0–37
Males	14.7	7.3	14.0	0–29
Females	16.6	7.3	17.0	2–37
VAS_mean_ score (*n* = 84)	4.3	2.1	4.0	1–9
SF-36 subscale (*n* = 84)				
Physical component score (PCS)	39.7	9.3	40.4	14.1–61.9
Physical functioning (PF)	69.6	20.9	72.5	5–100
Role-physical (RP)	43.0	42.6	29.2	0–100
Bodily pain (BP)	51.4	18.7	52.0	0–100
General health (GH)	65.6	24.7	67.0	0–100
Mental component score (MCS)	52.2	9.8	54.4	24.1–67.2
Vitality (VT)	58.0	23.6	60.0	0–100
Social functioning (SF)	80.8	22.5	87.5	25–100
Role-emotional (RE)	69.7	39.7	100.0	0–100
Mental health (MH)	76.4	16.6	80.0	20–100

**Table 3:** Pearson's correlation coefficients^†^ (*r*) between MFPDI-DK scores and SF-36 subscale scores (*n* = 84).

	MFPDI-DK_mean_	MFPDI-DK_day1_	MFPDI-DK_day2_
VAS	0.42*	0.41*	0.41*
SF-36 PCS	−0.66*	−0.66*	−0.63*
SF-36 PF	−0.69*	−0.70*	−0.65*
SF-36 RP	−0.57*	−0.56*	−0.48*
SF-36 BP	−0.52*	−0.52*	−0.50*
SF-36 GH	−0.46*	−0.44*	−0.46*
SF-36 MCS	−0.43*	−0.41*	−0.43*
SF-36 VT	−0.59*	−0.59*	−0.57*
SF-36 SF	−0.48*	−0.46*	−0.48*
SF-36 RE	−0.37*	−0.37*	−0.35*
SF-36 MH	−0.49*	−0.47*	−0.50*

^†^ *r* = 0: no correlation, *r* = + 1/(−1): perfect positive/negative correlation.

*Significant correlation, *P* < 0.001.

PCS*:* physical component summary score, PF: physical function, RP: role-physical, BP: bodily pain, GH: general health, MCS: mental component summary score, VT: vitality, SF: social function, RE: role-emotional, and MH: mental health.

**Table 4:** Agreement between test-retest scores of MFPDI-DK.

	Mean MFPDI-DK score (SD)			ICC score (95% CI)
	First assessment	Second assessment	Mean Difference (SD)	
MFPDI_total_ (*n* = 84)	15.8202381 (7.44)	15.8214286 (7.33)	0.0011905 (3.01)	0.92 (0.88–0.95)

SD: standard deviation, ICC: intraclass correlation coefficient, and CI: confidence interval.
